# Molecular mechanisms of mitochondrial quality control

**DOI:** 10.1186/s40035-025-00505-5

**Published:** 2025-09-01

**Authors:** Wensheng Li, Yuran Gui, Cuiping Guo, Yuting Huang, Yi Liu, Xuan Yu, Huiliang Zhang, Jianzhi Wang, Rong Liu, Yacoubou Abdoul Razak Mahaman, Qiuhong Duan, Xiaochuan Wang

**Affiliations:** 1 Institutes of Biomedical Sciences, School of Medicine, Hubei Key Laboratory of Cognitive and Affective Disorders, Jianghan University, Wuhan, 430056 China; 2 Department of Pathophysiology, School of Basic Medicine, Key Laboratory of Education Ministry/Hubei Province of China for Neurological Disorders, Tongji Medical College, Huazhong University of Science and Technology, Wuhan, 430030 China; 3 Tongji Hospital, Tongji Medical College, Huazhong University of Science and Technology, Wuhan, 430030 China; 4 Co-Innovation Center of Neuroregeneration, Nantong University, Nantong, 226001 China; 5 Hubei Provincial Key Laboratory of Kidney Diseases Occurrence and Intervention, School of Medicine, Hubei Polytechnic University, Huangshi, 435003 China; 6 Department of Biochemistry and Molecular Biology, School of Basic Medicine, Huazhong University of Science and Technology, Wuhan, 430030 China

**Keywords:** Mitochondria, Mitochondrial quality control, Mitochondrial homeostasis, Evidence-based therapies, Mitochondrial diseases

## Abstract

Mitochondria produce adenosine triphosphate (ATP), the main source of cellular energy. To maintain normal function, cells rely on a complex mitochondrial quality control (MQC) system that regulates mitochondrial homeostasis, including mitochondrial dynamics, mitochondrial dynamic localization, mitochondrial biogenesis, clearance of damaged mitochondria, oxygen radical scavenging, and mitochondrial protein quality control. The MQC system also involves coordination of other organelles, such as the endoplasmic reticulum, lysosomes, and peroxisomes. In this review, we discuss various ways by which the MQC system maintains mitochondrial homeostasis, highlight the relationships between these pathways, and characterize the life cycle of individual mitochondria under the MQC system.

## Introduction

Mitochondria are the epicenter of cellular energy production. Mitochondrial functions extend beyond mere metabolic functions and include a wide range of fundamental biological processes such as apoptosis, oxidative stress, signal transduction, and the cell cycle [1–3]. Disrupted mitochondrial homeostasis is implicated in the development of neurodegenerative disorders and metabolic diseases such as diabetes, obesity, and hypertension, exerting widespread effects on cellular function [4, 5].

Cells possess a complex and intricate mitochondrial quality control (MQC) system to regulate mitochondrial homeostasis, including clearance of damaged mitochondria, mitochondrial DNA repair, and maintenance of mitochondrial membrane potential. The MQC system encompasses several mechanisms to regulate mitochondrial homeostasis. The mitochondrial dynamics and motility serve as the foundation of MQC [6]. Mitochondrial fusion promotes functional complementation between damaged mitochondria [7], mitochondrial fission enables separation of damaged mitochondrial components [8], and mitochondrial dynamic localization ensures rapid and efficient energy production [9]. Mitochondrial biogenesis increases mitochondrial mass in response to increased energy demand to meet the cell’s energy needs, and this involves both mitochondrial biosynthesis and fission [10]. Reactive oxygen species (ROS) clearance mitigates mitochondrial oxidative damage [11, 12]. The mitochondrial protein quality control system ensures prompt degradation of damaged proteins, thereby maintaining mitochondrial protein homeostasis [13].

Mitochondria are susceptible to damage from diverse stresses, during which cells can initiate various MQC pathways to ensure proper mitochondrial functioning and prevent accumulation of dysfunctional mitochondria. This process involves the collaboration of multiple organelles, such as the endoplasmic reticulum (ER) [14, 15], lysosomes [16, 17], and peroxisomes [18, 19]. Proteases and proteasomes participate in mitochondrial protein quality control. Mitochondrial fusion, intercellular transfer of mitochondria [20], mitochondrial-derived vesicles (MDVs) [18], mitocytosis [21], and mitophagy are involved in the MQC pathways. Mitophagy is further divided into ubiquitin-dependent mitophagy [22], receptor-dependent mitophagy [23], and non-receptor-dependent mitophagy [24].

Here, we review the different aspects and components of the MQC system, the crosstalk between different components, and the mitochondrial life cycle, and then discuss future perspectives in the field of mitochondrial dysfunction conditions.

## Mechanisms underlying the maintenance of healthy mitochondria

### Mitochondrial dynamics and motility as the foundation of MQC

Alterations in mitochondrial morphology and subcellular localization are primarily influenced by mitochondrial dynamics, including mitochondrial fission and fusion as well as mitochondrial motility, such as mitochondrial recruitment and redistribution [25–27]. Mitochondrial fusion can enhance the ATP production capacity and promote functional complementation between damaged mitochondria. Mitochondrial fission can promote the generation of distinct functional mitochondrial units by partitioning the mitochondrial network, while also allowing for the dissociation of dysfunctional mitochondrial components. Thus, mitochondrial dynamics and motility are crucial for maintaining a homogeneous and healthy population of mitochondria within cells [6].

#### Mitochondrial fission and fusion in mitochondrial morphology and function

Mitochondrial fission can isolate damaged mitochondria, promoting mitophagy, whereas mitochondrial fusion can buffer acute damage within mitochondria by mixing healthy and impaired components of mitochondria, including mitochondrial DNA (mtDNA), proteins, and other matrix contents [7], thus maintaining mitochondrial function. Hyperfusion of mitochondria leads to the formation of an interconnected mitochondrial network, resulting in increased ATP production and reduced ROS [28, 29]. Abnormal fusion may also cause aberrant mtDNA copy numbers [30, 31]. Mitochondrial dynamics plays a critical role in cell-fate decision and in controlling mtDNA levels and distribution [31]. For example, overexpression of the inner mitochondrial membrane (IMM) protein mitochondrial fission process 1 (MTFP1), which negatively regulates IMM fusion, decreases mtDNA copy number, while *MTFP1* silencing or knockout increases the mtDNA copy number [31]. The same study further showed that MTFP1 is not only involved in the basal mtDNA degradation but also the selective disposal of damaged nucleoids, suggesting that fusion might decrease the degradation of mtDNA, resulting in increased copy number of mtDNA. Consistently, another study revealed that mitochondrial fusion might increase the mtDNA copy number by maintaining the stoichiometry of the protein components of the mtDNA replisome [32]. Additionally, fusion induces mtDNA synthesis by facilitating ROS-triggered, recombination-mediated replication [33]. Abnormal mitochondrial fusion is accompanied by reduced mtDNA copy number and a loss of mitochondrial membrane potential ($\Delta \Psi {\text{m}}$). This may result from impaired mtDNA distribution during fusion-fission cycles and compromised oxidative phosphorylation efficiency due to disrupted cristae architecture [30, 34]. These together suggest that fusion might increase the mtDNA copy via various mechanisms.

Conversely, excessive fission leads to fragmentation of the mitochondrial network and mitochondrial dysfunction [6, 35]. Interestingly, both mitochondrial fission and fusion are necessary for the thermogenic reaction of adipocytes. Fission promotes mitochondrial uncoupling respiration for thermogenesis [36], while deletion of mitochondrial fusion protein mitofusin 2 (MFN2) leads to thermogenic dysfunction [37]. These processes are fundamental for cell function and are regulated at multiple levels.

Three members of the dynamin family, MFN, optic atrophy 1 (OPA1), and dynamin-related protein 1 (DRP1), play a critical role in mitochondrial fission and fusion. Their functions are regulated by various adaptor proteins and regulatory processes on the mitochondrial surface.

Yeast is a commonly used model organism for studying mitochondria. Mitochondrial fusion-related proteins in yeasts fall into two categories: those that mediate IMM fusion, such as Mgm1 [38] (homologous to OPA1 in humans [39]), and those that mediate outer mitochondrial membrane (OMM) fusion, including Fzo1 and Marf [40] (homologous to MFN1 and MFN2 in humans [41, 42]). The critical protein responsible for mitochondrial fission, Dnm1 (homologous to DRP1 in humans [35]), originates from the cytoplasm and exhibits cyclical movement between the outer mitochondrial membrane and the cytoplasmic environment [43].

Mitochondrial fusion begins with contacts between adjacent mitochondria, followed by sequential fusion of OMM and IMM [44]. OMM fusion is regulated by MFN1 and MFN2. The two proteins have partially distinct roles, as MFN1 deficiency leads to uniformly sized spherical or short tubular mitochondria, while MFN2 knockout leads to various sizes of mitochondria [42]. During mitochondrial fusion, MFN proteins on the same mitochondrion form oligomers, while the MFN protein complexes between adjacent mitochondria form connections, thus increasing the contact between adjacent mitochondria [45, 46]. Subsequent GTP hydrolysis drives the conformational change of MFN and mediates OMM fusion. IMM fusion is driven by membrane potential. In the case of localized loss of membrane potential, OMM fusion can occur independently of IMM fusion [47]. This indicates that mitochondrial inner and outer membrane fusion may be regulated by the same upstream signal, with IMM fusion not interfering with OMM fusion, which explains why the matrix compartment in larger mitochondrial networks sometimes appears to be discontinuous. In addition, proteins such as MSTO1, MTCH2, SLC25A50, SLC25A46, ARL2, ELMOD2, and FBXL4 participate in the regulation of mitochondrial fusion [6]. It is worth noting that proteasomes may be involved in the last step of mitochondrial fusion [16].

Mitochondrial fission is initiated upon engagement of mitochondria with the ER, leading to ER tubules encircling the division site on mitochondria. This is facilitated by Spire-type actin nucleation factor 1C (Spire1C) and ER protein inverted formin-2 (INF2). The DRP1 protein is then recruited to the division site and oligomerized into rings, a process orchestrated by several OMM proteins, including mitochondrial fission 1 (Fis1), Mitochondrial fission factor (Mff), mitochondrial dynamics proteins (MiD) 49, and MiD51. The GTPase activity of DRP1 drives a series of conformational changes in the oligomeric rings, culminating in the scission of the mitochondrial membrane. Non-muscle myosin isoforms (NMIIA, NMIIB, and NMIIC) supply the force required for mitochondrial contraction. Myosin Va, an actin-based motor protein, interacts with Spire1C and DRP1 to facilitate mitochondrial fission, while the actin-depolymerizing protein Cofilin1 negatively modulates this process [6]. Notably, lysosomes contribute to the tagging of the sites of mitochondrial fission [48].

The processes of mitochondrial fusion and fission are regulated by various post-translational modifications such as ubiquitination, phosphorylation, and proteolytic cleavage of relevant proteins [35]. For example, the proteolytic cleavage of the long isoform of OPA1 (L-OPA1) generates the short isoform S-OPA1, which contributes to a shift towards mitochondrial fission [49]. Interestingly, cardiolipin (CL) on the IMM promotes fusion, while CL on the OMM promotes fission. High glucose conditions promote mitochondrial fission through O-GlcNAcylation modification [50]. Protein kinases such as ERK1/2, glycogen synthase kinase 3 beta (GSK3β), AKT, and PKA regulate mitochondrial fusion or fission through phosphorylation of implicated proteins [6, 35]. In addition, MIB, a member of the medium-chain dehydrogenase protein superfamily, selectively inhibits the function of MFN1 [51]. K63-ubiquitinated MFN1 promotes mitochondrial fusion by stabilizing oligomers [16], while K48-ubiquitinated MFN1 induced by Parkin promotes its degradation [52]. Pharmacological studies have identified several small-molecule compounds that regulate mitochondrial fission/fusion dynamics through targeting of core regulatory proteins. For example, Mdivi-1 specifically inhibits DRP1 [53], whereas Dynasore, initially developed as a dynamin GTPase inhibitor to block endocytosis, was found to regulate mitochondrial fission by inhibiting DRP1 [54].

#### Mitochondrial transport in mitochondrial dynamic localization

**Fig. 1:**
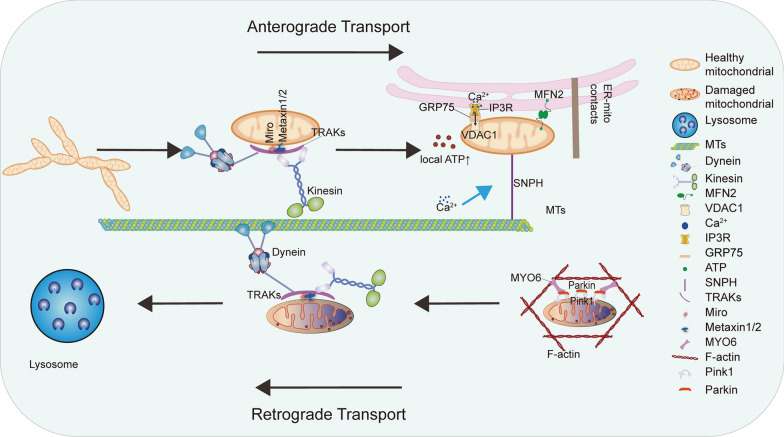
Mitochondrial dynamic localization. Anterograde transport is primarily mediated by Kinesin, and retrograde transport is mainly dependent on Dynein, with the participation of microtubules and TRAKs/MIRO/Metaxin1/2. Mitochondrial anchoring relies on SNPH and ER-mito contacts. Damaged mitochondria can be encapsulated by an F-actin cage with MYO6 and transported to the lysosome via retrograde transport

Mitochondrial dynamic localization plays a crucial role in maintaining normal cellular energy metabolism and function, especially in high-energy-demanding cells, such as neurons and muscle cells. It involves the translocation of mitochondria to specific cellular regions with high energy demand, enabling efficient energy production and supply [9, 27]. This process is also essential in response to cellular stress, where rapid mitochondrial transfer is required to meet the cell’s energy demand [55]. The dynamic localization of mitochondria is influenced by various cellular factors, including cytoskeletal transport, particularly microtubule-dependent transport of mitochondria [56], Ca^2+^ signaling [57], ER assistance [58], as well as mitochondrial fission and fusion [35] (Fig. 1).

Mitochondrial dynamic localization mainly relies on the mitochondrial transport mechanism [27, 55]. Mitochondrial transport requires the involvement of the outer mitochondrial membrane Rho GTPase (Miro), the trafficking kinesin-binding proteins (TRAKs) (Milton in *Drosophila*), the driving proteins KIF, and the cytoplasmic motor protein dynein. Kinesin family member 5 (KIF5) is the main driving protein that transports mitochondria in an anterograde direction to dendrites and axons, while dynein proteins mediate the retrograde transport of mitochondria to the cell body [59]. The OMM protein Metaxin1/2 promotes mitochondrial transport by forming a complex with Miro and TRAK [56, 60], and the coordinated recruitment and activation of dynein and KIF drives the bidirectional transport accordingly [61]. Miro connects KIF5 via TRAKs or the *Drosophila* ortholog Milton, thereby linking KIF5 to mitochondria [62, 63]. Metaxin1/2 also contributes to mitochondrial transport by forming a complex with MIRO and KIF5 in *C. elegans* [60]. KIF5 and dynein coordinate opposing movement activities and drive bidirectional mitochondrial transport [64]. Syntaphilin (SNPH) is a mitochondrial anchoring protein that is involved in positioning mitochondria at high-energy-consuming sites to provide stable energy [65]. The AKT-PAK5-SNPH signaling axis regulates mitochondrial anchoring [66]. However, how mitochondria are precisely transported to their destination remains an interesting question. Further research is needed to reveal how energy demands are converted into signals that induce the recruitment of mitochondria OMM.

Mitochondrial dynamic localization can be affected under various conditions. For instance, elevated extracellular glucose levels reduce mitochondrial migration ability through O-GlcNAcylation of Milton [67]. During synaptic activation in neurons, mitochondrial transport is hindered by the influx of glutamate and Ca^2+^ via Miro. This triggers repositioning of mitochondria at synaptic sites that require ATP [57]. Notably, both Mfn1 and Mfn2 interact with Miro and Milton, and loss of Mfn2 alone leads to defective neuronal mitochondrial transport [68]. It has been shown that ER and mitochondria are preferentially transported along microtubules with acetylated microtubule proteins. ER stress drives ER and mitochondrial migration to the perinuclear region, resulting in increased contact points between the two organelles [58], which may limit the dynamic localization of mitochondria.

### Mitochondrial biogenesis ensures enough functional mitochondria for maintaining cellular energy

Mitochondrial biogenesis is the process of generating new mitochondria through the growth and division of existing mitochondria. It plays a pivotal role in maintaining cellular energy homeostasis and meeting metabolic demands. Importantly, damaged mitochondrial components can be replaced via mitochondrial biogenesis [10]. This process involves a symmetrical mitochondrial division, resulting in the formation of new mitochondria with normal membrane potential [69]. Mitochondrial biogenesis is a compensation for effective quality control during asymmetric fission (e.g., mitophagy). Mitochondrial biogenesis and mitophagy ensure the removal of damaged and/or superfluous mitochondria, as well as the balanced generation of new mitochondria [70]. Impaired mitochondrial biogenesis contributes to mitochondrial dysfunction in many diseases [10, 71–73].

The process of mitochondrial biogenesis is tightly regulated by a variety of transcriptional co-activators and nuclear transcription factors based on the NAD^+^/NADH ratio, the AMP/ATP ratio, or the acetyl-Coenzyme A (acetyl-CoA) levels [74]. Notably, peroxisome-proliferator-activated receptor gamma co-activator-1 alpha (PGC-1α) and nuclear respiratory factor 2 (NRF2) are two major transcriptional co-activators that control the expression of nuclear-encoded mitochondrial genes [75]. Activated PGC-1α enhances the activity of some DNA-binding transcription factors, such as NRF1/2, peroxisome-proliferator-activated receptors PPARα, PPARδ, PPARγ, and estrogen-related receptors ERRα, ERRβ, and ERRγ, thereby promoting transcription of mitochondrial regulatory proteins [69].

**Fig. 2:**
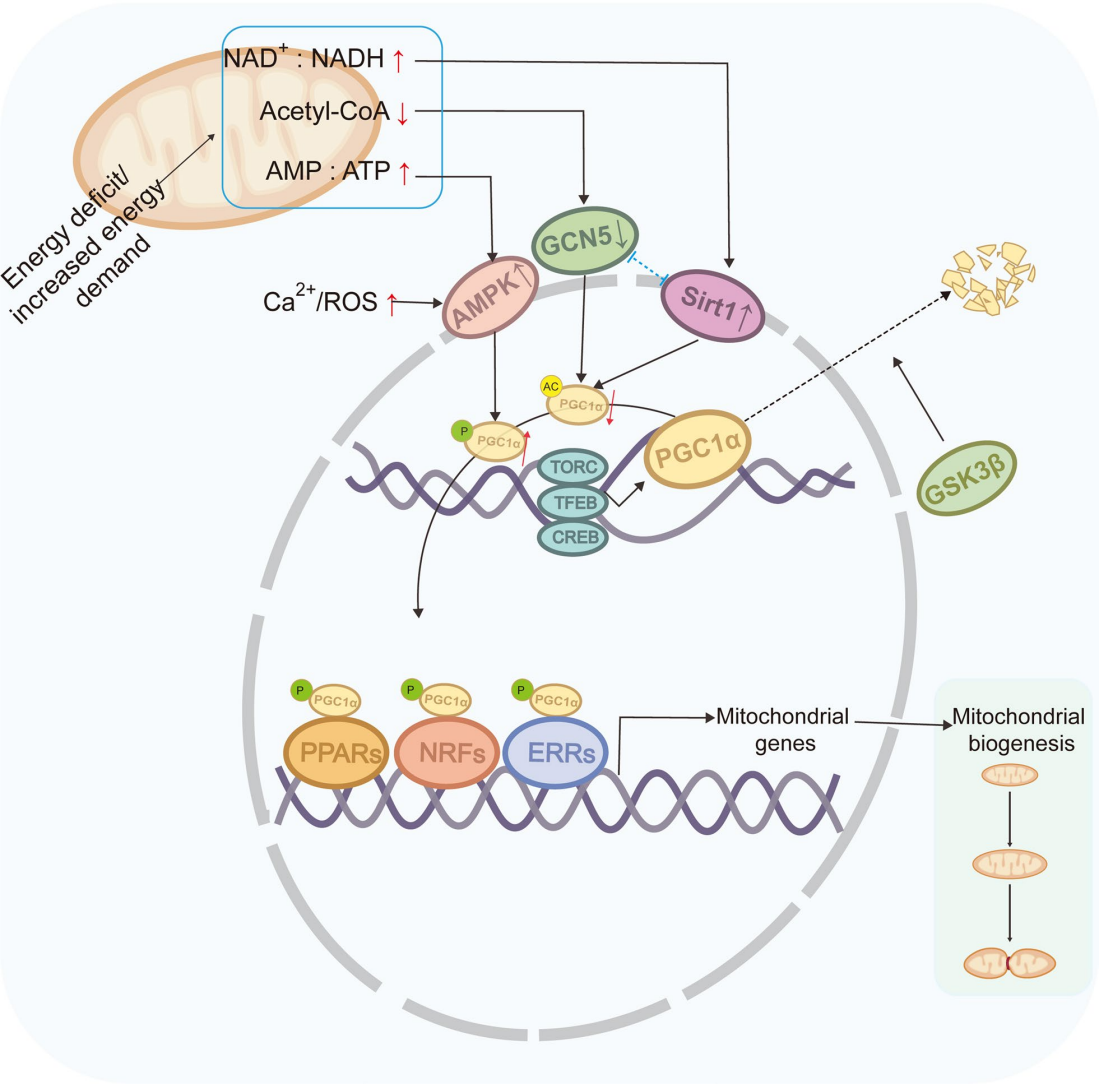
Regulation of mitochondrial biogenesis. Energy deficits or increased energy demands, such as elevated NAD^+^/NADH ratio, AMP/ATP ratio, Ca^2^⁺ or ROS levels, and decreased acetyl-CoA levels, activate upstream signaling pathways such as AMPK, Sirt1, and downregulate GCN5, reducing PCK1 acetylation levels while enhancing its phosphorylation levels. Activated PGC1α elevates PPARs, NRFs, and ERRs to induce the expression of mitochondrial genes. Transcriptional regulators TORC, TFEB, and CREB activate PGC-1α transcription, while GSK3β promotes PGC-1α degradation via phosphorylation

The expression and activity of PGC-1α are regulated by multiple signaling molecules (Fig. 2). For example, transcription factor EB (TFEB) and cAMP response element-binding protein (CREB) promote transcription of PGC-1α [76, 77], the transducer of regulated CREB-binding proteins (TORCs) modulate PGC-1α promoter activity [78], whereas GSK3β inhibits PGC-1α transcription by inactivating TFEB and promotes PGC-1α degradation via phosphorylation [79, 80]. AMP-activated protein kinase (AMPK) is activated in response to increases in the AMP/ATP ratio, ROS, and Ca^2+^ signaling [81, 82], and then phosphorylates and activates PGC-1α [83]. In addition, PGC-1α is acetylated and inhibited by general control nonrepressed 5 (GCN5), which requires acetyl-CoA as a substrate for the acetylation reaction. Elevated acetyl-CoA level promotes the acetylation activity of GCN5 [84], while the deacetylase Sirt1 activates PGC-1α by counteracting the acetylation activity of GCN5 upon an increase in the NAD^+^/NADH ratio [85, 86]. Taken together, mitochondrial biogenesis is a complex and highly regulated mechanism involving several factors that are activated upon the energy requirements of the cell.

### Clearance of damaged and excess mitochondria

Mitochondria are the primary location of cellular oxidative phosphorylation, a process that produces free radicals and renders the organelle vulnerable to oxidative damage. Failure to efficiently eliminate damaged mitochondria can result in impairment of normal cellular functions. Mitophagy is the main process that removes dysfunctional or excess mitochondria. However, other pathways, including intercellular mitochondrial transfer [20], MDVs [18], and mitocytosis [21], have also been identified.

#### Mitophagy-mediated removal of excess and damaged mitochondria

**Fig. 3:**
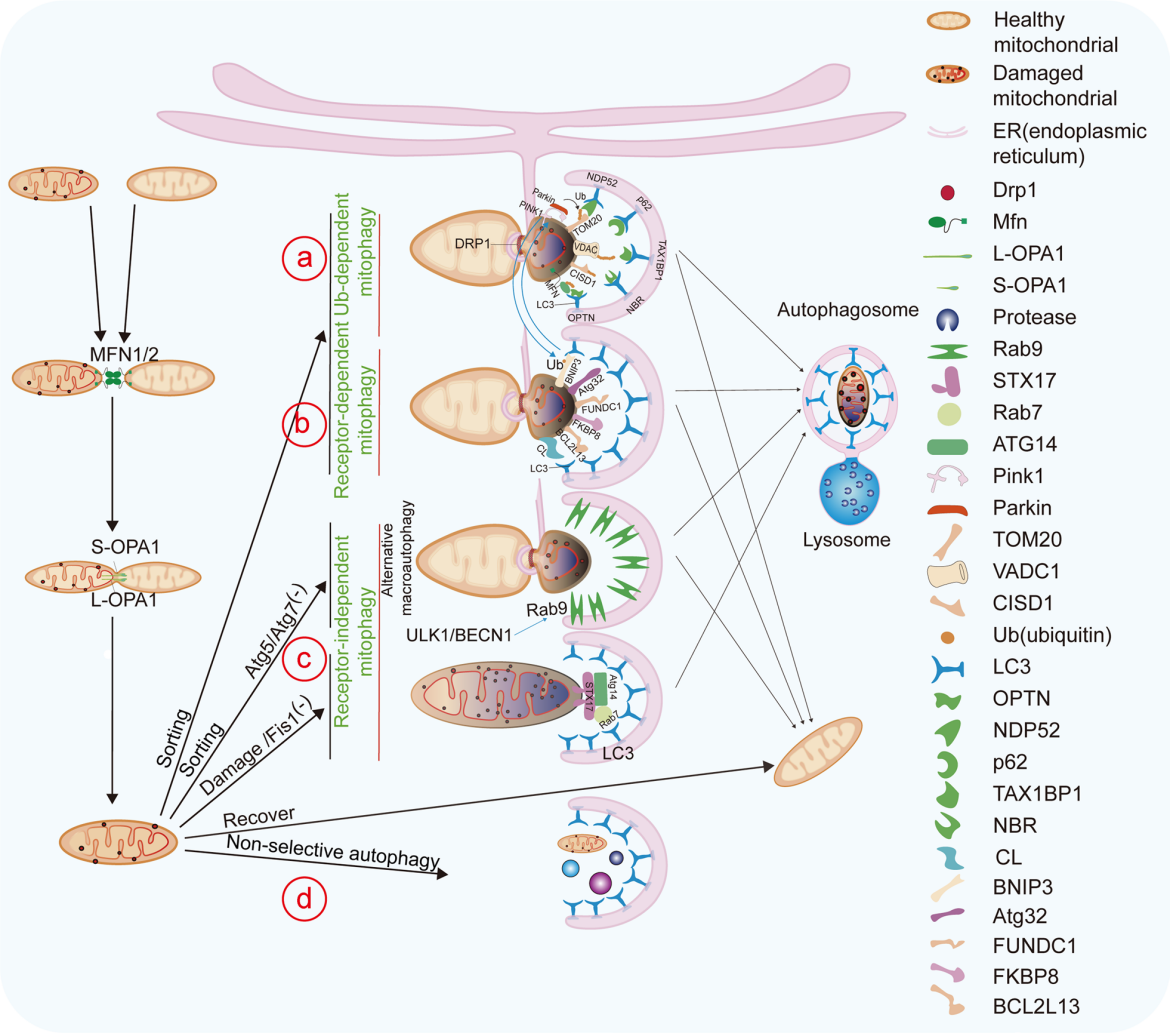
Mitophagy maintains mitochondrial quality control. **a** Ubiquitin-dependent mitophagy: Pink1 accumulates on the outer mitochondrial membrane of damaged mitochondria and recruits Parkin. Parkin ubiquitinates outer membrane proteins (e.g., CISD1, TOMM20, VDAC, MFN), enabling autophagy adaptors (e.g., OPTN, TAX1BP1, p62, NDP52, NBR) to interact with LC3 and initiate mitophagy. **b** Receptor-dependent mitophagy: damaged mitochondria are recognized by specific receptors, such as FUNDC1, NIX/BNIP3, CL, Atg32, FKBP8, and BCL2L13, which recruit LC3 via their LC3-interacting region (LIR) to initiate mitophagy. **c** Receptor-independent mitophagy. **d** Non-selective mitophagy through macroautophagy

Mitophagy is the major pathway for the clearance of damaged mitochondria and is essential in processes such as germ cell development [87], retinal ganglion cell differentiation [88], erythrocyte maturation, cardiac cell maturation, and M1 macrophage polarization [88]. Depending on the specific cellular contexts, mitophagy can be promoted in response to various stimuli, such as oxidative stress, calcium overload, and membrane potential collapse [89], to regulate mitochondrial quantity/quality and maintain energy metabolism [23]. Mitophagy is a process of macroautophagy that eliminates excess and damaged mitochondria through lysosomal degradation [17] (Fig. 3). Macroautophagy can be classified into selective and non-selective forms [90]. The selective mitophagy can be ubiquitin-dependent and ubiquitin-independent [22]. The ubiquitin-independent mitophagy can be further categorized into receptor-dependent [23] and receptor-independent [91].

##### Ubiquitin-dependent mitophagy

The ubiquitin-dependent mitophagy is controlled by the PTEN-induced putative kinase 1 (Pink1)–Parkin pathway. At physiological conditions, Pink1 translocates across the OMM via the translocase of the outer membrane (TOM) complex and penetrates the IMM aided by the inner membrane potential and the translocase of the inner mitochondrial membrane (TIM) complex [92]. After translocation, Pink1 is cleaved by the mitochondrial processing peptidase and presenilin-associated rhomboid-like protease (PARL) to generate cleaved Pink1 (c-Pink1) [92]. PACT, a low-activity form of PARL generated by proteolytic cleavage, is necessary for Pink1/Parkin-mediated mitophagy [93]. The N-terminus fragment of c-Pink1 is normally unstable and, after retro-translocation from the mitochondria to the cytosol, is recognized by E3 ubiquitin ligases UBR1, UBR2, and UBR4 through the N-terminal region and subsequently degraded by the proteasome [94]. However, some c-Pink1 can remain relatively stable by binding to molecular chaperones, and thus activate prosurvival pathways and play an opposite role compared to full-length Pink1 in mitophagy [95, 96].

When mitochondria become defective, the mitochondrial membrane potential (ΔΨm) is decreased, and the depolarized mitochondria block the opening of the TIM23 channel [97], which prevents Pink1 cleavage and rapidly stabilizes proteins on the TOM complex. The TOM complex promotes Pink1 dimer formation, resulting in PINK1 autophosphorylation and Parkin recruitment [98]. Pink1 selectively forms a 700 kDa complex with the TOM complex on depolarized mitochondria, with TOM20, TOM22, TOM40, and TOM70 participating in complex formation [99]. Pink1 accumulating on the OMM recruits and phosphorylates ubiquitin molecules as well as Parkin, thereby activating its E3 ubiquitin ligase activity [100] and leading to the ubiquitination of OMM proteins such as CISD1, TOM20, VDAC, and MFN [101]. In addition to Parkin, several other ubiquitin E3 ligases, such as Gp78, SMURF1, SIAH1, MUL1, and ARIH1, also participate in mitophagy regulation, recruiting ubiquitin-binding mitophagy adapter proteins such as OPTN, TAX1BP1 (Tax1 binding protein 1), SQSTM1 (p62), CALCOCO2/NDP52 (NBR1 Domain Containing 52), and NBR [23]. A model proposes that the initial recruitment and activation of Parkin require protein ubiquitination mediated by the ubiquitin ligase MITOL/MARCH5 and subsequent Pink1-mediated phosphorylation [102]. Multiple stress-related transcriptional regulatory factors are involved in the transcriptional regulation of Pink1 in the process of Pink1/Parkin-mediated mitophagy. For instance, Foxo3a, NFκb, NRF2, and PTEN upregulate the expression of Pink1 in cells, while ATF3 and P53 downregulate Pink1 expression [96].

##### Receptor-dependent mitophagy

The currently known mitophagy receptors include FUNDC1, BCL2L13, FKBP8, ATG32, and BNIP3 [22]. In addition, cardiolipin, which translocates to the OMM after mitochondrial depolarization, also participates in the initiation of mitophagy [103]. Mitophagy receptors bind to LC3 during mitochondrial stress. NIX and BNIP3 can regulate Parkin recruitment to maintain mitochondrial homeostasis [104, 105], and Parkin, in turn, promotes the binding of BNIP3 to LC3 through ubiquitination [106]. These findings suggest that there may be mutual promotion between the receptor-dependent and the ubiquitin-dependent (Pink1–Parkin) mitophagy pathways.

##### Receptor-independent mitophagy

In the absence of the OMM protein Fis1, the synaptic fusion protein 17 (Syntaxin 17, STX17) is dynamically transported from the ER to the mitochondria and undergoes self-oligomerization, recruiting ATG14, Rab7, and other factors to activate mitophagy [107]. Additionally, there is a unique mitophagy pathway independent of ATG5 or ATG7, known as alternative macroautophagy. The formation of autophagosomes in alternative macroautophagy begins in the trans-Golgi network and depends on factors such as RAB9, WIPI3 (WD repeat domain phosphoinositide interacting 3), and upstream autophagy factors Unc-51 like autophagy activating kinase 1 (ULK1) and BECN1 (Beclin 1) [24, 91].

#### Mitochondrial intercellular transfer: utilizing other cells to remove damaged and excess mitochondria

Studies have shown that mitochondria can detach from the cell they originate from through various mechanisms including tunneling nanotubes (TNTs), microvesicles, exosomes, and cytoplasmic fusion [20, 108], and be taken up by other cells. This results in intercellular transfer and aids in MQC. In mATG8-deficient cells, mitochondria can be released into the extracellular space and then be cleared through a secretory autophagy pathway known as autophagic secretion of mitochondria [109]. In retinal ganglion cells, axonal mitochondria are shed through large protrusions and degraded by adjacent astrocytes [110]. Neurons can release damaged mitochondria for transfer to astrocytes, and in turn, astrocytes can release healthy mitochondria for uptake by neurons, a process that potentially aids in post-stroke neuronal recovery [111]. Intercellular transport of mitochondria helps maintain mitochondrial homeostasis within cells. However, while numerous studies have demonstrated that healthy mitochondria can undergo one-way intercellular transfer via TNTs [112–118], there are also reports suggesting that mitochondria may undergo bidirectional transfer through TNTs [119]. It remains unclear whether damaged mitochondria can be transferred to other cells via TNTs for clearance. Future research in this field may open up new avenues for treating mitochondria-related diseases.

#### MDVs and vesicles derived from the IMM (VDIMs): the first line of defense against oxidative stress

MDVs are small vesicles derived from mitochondria that carry mitochondrial cargoes. VDIMs are formed by IMM herniation through pores formed by the voltage-dependent anion channel (VDAC1) in the OMM. As the critical mechanism in MQC, MDVs and VDIMs serve as the first line of defense against oxidative stress by selectively transferring and degrading damaged mitochondrial materials before mitophagy occurs.

**Fig. 4:**
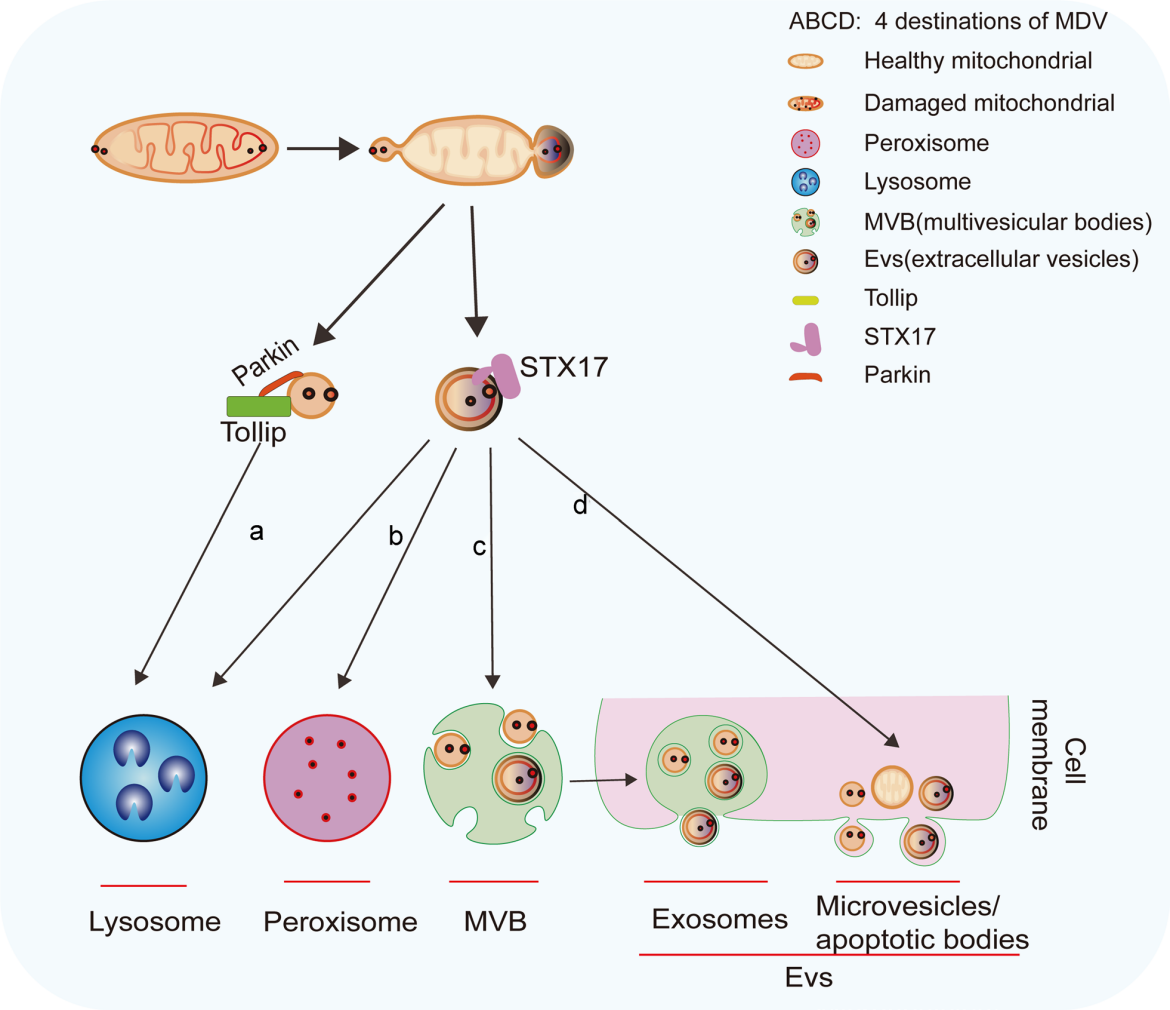
Destinations of mitochondrial-derived vesicles (MDVs) for mitochondrial quality control. MDVs serve as a quality control mechanism to maintain mitochondrial homeostasis, and have the following four destinations. **a** Lysosomes. Single-membrane MDVs are included in lysosomes through synergistic functions of the Toll-interacting protein (Tollip) and Parkin. Some double-membrane MDVs are transported via STX17 to lysosomes. **b** Peroxisome. Specific MDVs interact with peroxisomes to facilitate detoxification of reactive oxygen species (ROS). **c** Multivesicular bodies (MVBs). MDVs are incorporated into MVBs under physiological conditions. **d** Extracellular vesicles (EVs). MDVs can be secreted extracellularly via exosomes or released as microvesicles and apoptotic bodies, contributing to intercellular communication or removal of mitochondrial debris

MDV biogenesis can be triggered by ROS, Pink1, and DRP1. Importantly, dysregulation of MDV degradation can disrupt metabolite clearance and immune responses, contributing to diseases such as neurodegeneration, cardiovascular diseases, and cancer [18]. MDVs bud from mitochondria as single-membrane or double-membrane vesicles [120] that selectively incorporate damaged portions of mitochondria. These damaged parts can be sorted for degradation in late endosomes/lysosomes, targeted for peroxisomes, or even transported outside the cell [120, 121] (Fig. 4). Different MDV subtypes with specific pathways and destinations have been identified [122, 123]. For instance, Pink1 can initiate MDV formation in response to stress, and the single-membrane MDVs are synergistically transported to lysosomes through the Toll-interacting protein (Tollip) and Parkin. Double-membrane MDVs are transported via the SNARE family member STX17. Mthsp70-positive MDVs are another type of MDV that are transported to the extracellular space via sorting nexin 9 (SNX9) and multivesicular bodies under normal physiological conditions or degraded in lysosomes under stress. However, MDVs carrying MAPL (mitochondria-anchored protein ligase) are transported to peroxisomes with the assistance of Vps35 (vacuolar protein sorting 35) [18]. Interestingly, live-cell imaging showed that MDVs can release mitochondrial contents into extracellular vesicles [124] in the form of exosomes, microvesicles, and apoptotic bodies [20, 124, 125]. Further, macrophages in adipose tissue, cardiac tissue, and mesenchymal stem cells (MSCs) phagocytose these MDV-released mitochondria to maintain mitochondrial homeostasis [124, 126, 127]. Furthermore, damaged mitochondrial inner membrane can herniate through VDAC1 ligomerization-induced pores in the OMM, undergoes ESCRT-mediated VDIM formation, and is subsequently engulfed by adjacent lysosomes. VDIM formation is enhanced in mitochondria undergoing oxidative stress, suggesting their potential role in maintaining mitochondrial function [128].

In addition to mitochondrial clearance, MDVs can also facilitate communication and material transport between cells or organelles in eukaryotes. For example, MDVs can transfer superoxide dismutase 2 (SOD2) to macrophages, promoting hydrogen peroxide production [129]. Bcl2 may also be transferred by MDVs to the ER or severely damaged mitochondria to prevent unwanted apoptosis [130, 131]. Interestingly, toxic MDVs from adipocytes taken up by cardiomyocytes and fusing with their mitochondria may provide an effective way for cardio-protection [132]. However, there is no sufficient evidence that MDVs act as carriers for transporting metabolites between two organelles. Therefore, further research is needed to confirm this possibility.

#### Mitochondrial exocytosis: MQC mediated by migrasomes

Migrasomes are extracellular vesicular organelles produced by migrating cells [133]. During cell migration, contractile fibers pull from the rear to form migrasomes. When these fibers break, the vesicular structures detach and release cellular contents [134]. Subsequent investigations have identified that mild mitochondrial stress triggers the selective binding of migrasomes to damaged mitochondria at the cell periphery. As the cell migrates, mitochondria are released outside the cell through migrasomes, enabling quality control of mitochondria. This process is referred to as mitocytosis [21]. Mitocytosis prevents mitochondrial stress-induced damage to mitochondrial membrane potential and function, thus playing a crucial role in maintaining healthy mitochondria.

## Regulation of mitochondrial homeostasis

### Critical role of ROS clearance

Mitochondria are the primary site of ROS generation [135]. During oxidative phosphorylation, the electron transport chain (ETC) renders mitochondria the most susceptible organelles to ROS generation. Various factors within mitochondria facilitate ROS generation at disease states. For example, dysfunction of pro-apoptotic protein HTRA2 lowers the membrane potential and ATP production, thereby promoting ROS generation and causing premature aging [136, 137]. Abnormalities of the L-OPA1/S-OPA1 ratio or the loss of OPA1 can cause the swelling of cristae, leading to the displacement of respiratory super-complexes and promoting the generation of ROS [138–140]. Moreover, MFN2 deficiency promotes the opening of mitochondrial permeability transition pores, which also increases ROS production [141]. Although low levels of ROS may have physiological effects [142, 143], excessive ROS production can lead to damage to mtDNA [144], mitochondrial proteins [143, 145], and lipids [146, 147], as well as abnormalities in multiple signaling pathways [148]. Additionally, ROS release from mitochondria can trigger further ROS release from the neighboring mitochondria [149], highlighting the importance of ROS clearance systems in maintaining mitochondrial homeostasis.

ROS clearance is an important antioxidant defense system within the body, which involves the participation of peroxisomes [19]. The molecular components involved in ROS clearance include catalase (CAT), ascorbate peroxidase (APX), superoxide dismutase (SOD), glutathione reductase (GR), glutathione peroxidase (GPx), monodehydroascorbate reductase (MDHAR), dehydroascorbate reductase (DHAR), glutathione-S-transferase (GST), and non-enzymatic compounds such as ascorbic acid (AsA), α-tocopherols, and glutathione (GSH) [11, 12]. These components work synergistically to clear excess oxygen-free radicals, reduce oxidative damage, and maintain normal cellular metabolism and physiological function. Cellular autophagy [150] and mitochondrial proteases also play important roles. For instance, Complex I is degraded by the proteases LONP1 and CLPP during mitochondrial depolarization, thereby reducing ROS production [151].

There are also feedback regulatory mechanisms for ROS scavenging. For instance, ROS stimulates the movement of TFEB from the cytoplasm to the nucleus, where it is activated by dephosphorylation [152, 153]. In turn, TFEB-mediated activation of autophagy can promote lysosomal biogenesis and function, regulate autophagy proteins, and eliminate ROS [150].

### Mitochondrial protein homeostasis

Mitochondrial protein homeostasis (proteostasis) is critical for normal mitochondrial function. Mitochondrial proteins are vulnerable to oxidation and post-translational modifications [154] resulting from mitochondrial metabolism, which can affect protein import, signaling cascades, mitochondrial dynamics, and lipid biosynthesis [155]. Mitochondria possess a distinct protein quality control system to maintain protein homeostasis [13].

Mitochondrial proteinases and chaperones play a crucial role in processing precursor proteins entering mitochondria and eliminating misfolded or damaged proteins. The majority of mitochondrial proteins are synthesized as precursor proteins in the cytosol and transported into mitochondria via five distinct pathways: the classical presequence pathway and the other four pathways in which precursor proteins possess different kinds of internal targeting signals [97]. Mas1 is responsible for removing the pre-sequence of mitochondrial matrix proteins [156], while IMMP1L and IMMP2L cleave the hydrophobic stop-transfer signal of intermembrane space proteins [157]. Some proteins are additionally cleaved by Pcp1, generating both full-length and truncated forms [157]. Moreover, some mtDNA-encoded proteins also undergo cleavage by proteinases, including the N-terminal Met excision pathway [158]. Interestingly, there are three major classes of mitochondrial proteinase that maintain protein homeostasis after proteins enter mitochondria, including ATP-dependent proteases, such as mAAA (AFG3L2 and SPG7), iAAA (YEM1), LONP1, and ClpXP; ATP-independent proteases, like HTRA2 and ATP23; and oligopeptidases, such as PITRM1 [159].

In addition to mitochondrial proteases, the cytoplasm is also involved in the regulation of mitochondrial proteostasis. Mitochondrial-associated degradation (MAD) is the clearance of damaged mitochondrial proteins through the ubiquitin–proteasome system [160]. In the MAD process, damaged OMM proteins undergo ubiquitination by specific E3 ubiquitin ligases. The ubiquitinated substrates are then recognized by substrate-recruiting factors such as Vms1 and Doa1 [161], extracted from the OMM driven by the energy from ATP hydrolysis, and degraded by proteasomes [162]. The ribosome quality control mechanism is another pathway implicated in the regulation of mitochondrial proteostasis. Cells may produce faulty mitochondrial polypeptides that stall on ribosomes through elongation with a C-terminal alanine/threonine (CAT) tail by Rqc2 and are then imported into the mitochondria. The CAT tail facilitates peptide aggregation within the mitochondria, thereby promoting toxicity. The cytosolic protein Vms1 counteracts Rqc2 by releasing stalled peptides from the 60S ribosome, reducing the CAT tail, and facilitating their clearance [163].

Mitochondria contain a unique class of proteins, termed mitochondrial long-lived proteins (mt-LLPs). Despite the organelle’s highly dynamic nature, these proteins remain stable for months within its confined space and with limited molecular exchange. It has been proposed that mt-LLPs could provide lifelong structural stability to mitochondria, known as the dynamic stability of mitochondria [164].

In summary, the protein quality control mechanisms in mitochondria and cytoplasm collaborate to maintain mitochondrial proteostasis.

### mtDNA homeostasis

mtDNA homeostasis is currently a field of intense interest given the links between cytosolic mtDNA and innate immunity, specifically activation of pro-inflammatory signaling pathways. mtDNA homeostasis is important for mitochondrial and cellular functions. mtDNA encodes 13 subunits of the ETC, 22 tRNAs, and 2 rRNAs, so its integrity is essential for cellular energy production [165]. Unlike nuclear DNA, mtDNA lacks histones, replicates independently of the nuclear genome without proofreading, and is exposed to ROS generated during oxidative phosphorylation, rendering it highly susceptible to damage [166].

Damaged mtDNA fragments can be released into the cytosol through multiple mechanisms, depending on the stress level of mitochondria, cells, and the local microenvironment. The mechanisms include release through BAK-BAX megapores during apoptosis [167], mitochondrial permeability transition pores [168, 169], and ROS-induced oligomerization of VDAC1 on the mitochondrial outer membrane [170]. mtDNA released into the cytosol then activates the cGAS-STING pathway and innate immune responses [171], leading to aging [172], systemic lupus erythematosus, Parkinson’s disease, amyotrophic lateral sclerosis, etc. [170] [173].

mtDNA homeostasis involves replication, repair, selective degradation of damaged mtDNA, and quality control. First, mitochondria possess almost all DNA repair machineries for base excision repair, mismatch repair, and recombinational repair. The DNA repair nuclease, such as MRE11A, can function as a mitochondrial protector [174]. Chaperones such as HSP90-beta, HSP70, and mitochondrial transcription factor A (TFAM), protect the stability of mtDNA [175]. Furthermore, PARK2 and LRRK2 specifically take part in mtDNA maintenance [176]. Second, damaged mtDNA is degraded through mitochondrial autophagy (mitophagy) in most cells [30, 31, 177, 178]. Some studies have identified VDIMs [128] and SNX9-dependent MDVs [179] that encapsulate mtDNA, as novel pathways for mtDNA clearance. Recent studies found that the mtDNA can directly shuttle to a recycling organelle in a BAX-dependent manner [180]. In addition, when mtDNA fragments are released into the cytosol, the autophagy receptor TFAM facilitates selective degradation of damaged mtDNA through nucleoid-phagy pathways via the canonical endosomal-lysosomal pathway: RAB5 orchestrates early endosomal engulfment, while RAB7 governs subsequent trafficking to late endosomes and lysosomes (endolysosomes), where nucleoid constituents are enzymatically processed [181, 182].

### ER-mediated regulation of mitochondrial homeostasis

ER and mitochondria are connected by structural interfaces referred to as ER-mito contacts [14]. These contacts are implicated in lipid biosynthesis and transport [183], signal (ROS, protein kinases, calcium) propagation to mitochondria [184], organelle morphogenesis, including mitochondrial biogenesis, fission, autophagy, and inheritance [185, 186]. Some of the major complexes in ER-mito contacts include the VAPB (ER)-PTPIP51 (OMM) complex which plays a role in autophagy and lipid synthesis and transport, the ORP5 (ER)-PTPIP51 (OMM) complex involved in sterol transport, the BAP31 (ER)-FIS1 (OMM) complex related to cell death, the INF2 (ER)-Actin-Spire1C (OMM) complex involved in mitochondrial fission, the IP3R (ER)-GRP75-VDAC (OMM) complex involved in calcium transport, the MFN2 (ER)-MFN2/1 (OMM) complex involved in multiple processes, and the RRBP1 (ER)-OMP25 (OMM) complex with no known function [187]. In addition, TMBIM6 (transmembrane bax inhibitor motif containing 6) on ER interacts with VDAC1 on OMM and prevents its oligomerization, thus facilitating calcium transport [188].

The ER-mito contacts enhance mitochondrial fission through ER-localized INF2. IFN2 induces actin polymerization, driving initial mitochondrial constriction, which further induces Drp1 recruitment to mitochondria, facilitating secondary constriction [15]. This highlights the importance of ER in the regulation of mitochondrial morphology and dynamics. Moreover, ER also regulates mitochondrial transport and distribution. ER stress drives the microtubule-dependent redistribution of ER and mitochondrial networks to the perinuclear region, accompanied by an increase in ER-mito contacts [58].

ER-mito contacts are closely associated with Ca^2+^ transport. In cardiac myoblasts, an increase in cytoplasmic Ca^2+^ slows/stops mitochondrial movement by affecting the binding of mitochondria to microtubules. This Ca^2+^-associated halt in mitochondrial movement may, in turn, promote ER-mito contacts and Ca^2+^ release from ER [189]. Additionally, Miro, a mitochondrial transport regulator, also colocalizes with ER-mito contacts [190]. This colocalization suggests functional interplay: when mitochondrial transport stops, Miro may participate in the formation of ER-mito contacts, indicating potential competition between mitochondrial transport and ER-mito contact maintenance.

ER regulates mitophagy via ER-mito contacts, where Pink1-Parkin activation and ubiquitination events occur. Interestingly, loss of the VAPB (ER)-PTPIP51 (OMM) complex impairs autophagy [187]. PERK (protein kinase RNA-like endoplasmic reticulum kinase) is an ER stress sensor localized at ER-mito contacts that detects organelle stress and regulates mitophagy [191]. Evidence shows that PTPIP51, an ER-mito anchoring protein, suppresses Parkin-mediated mitophagy [192]. Conversely, the Pink1-Parkin-mediated degradation of MFN2 disrupts ER-mito contacts, facilitating mitophagy [193]. However, a recent study found that increased MFN2 acts as an ER-mito tethering antagonist that results in reduced ER-mito coupling [192]. ER-mito contacts may protect specific OMM proteins from the Pink1-Parkin-dependent degradation, thereby negatively regulating mitophagy [23]. In addition, mitochondrial Rho GTPase 1 (Miro1) is involved in the formation of ER-mito contacts, and the removal of Miro1 from depolarized mitochondria may aid in the clearance of damaged mitochondria through mitophagy [194]. Taken together, these findings suggest complex regulation of mitophagy by ER-mito contacts. While certain ER-mito contacts may be necessary for the initiation of mitophagy, removal of ER-mito contacts is required for the completion of mitophagy.

## Interconnections between distinct MQC pathways

MQC pathways are not distinct from one another but are rather interconnected. Activation of one MQC pathway does not simply promote or inhibit another MQC modality, but rather, these pathways can interact and cooperate under certain conditions to maintain mitochondrial homeostasis. The relationship between different MQC pathways may be further complicated by compensatory mechanisms.

### Relationships between mitochondrial dynamics

#### Interactions between mitochondrial fission and fusion processes

Mitochondrial fusion and fission are regulated by multiple proteins, including metalloendopeptidase YME1L1 and OMA1, which cleave OPA1 to generate a greater amount of S-OPA1, shifting the balance towards mitochondrial fission [49]. This regulatory mechanism determines the opposing nature of mitochondrial fusion and fission. However, when Drp1 is knocked out, the levels of Mfn1 and Mfn2 decrease by approximately 50% [195, 196]. This compensatory response may partially rebalance mitochondrial dynamics but is insufficient to prevent functional impairment in some cases. Additionally, live-cell imaging analysis revealed that mitochondria periodically fuse with each other, triggering asymmetrical fission that does not affect the time required for subsequent fusion [29]. However, the resulting daughter mitochondria often have different membrane potentials [30], and the defective portions generated by asymmetric division subsequently enter the mitophagy pathway [197]. MitoPLD, a lipid metabolism enzyme, is involved in the generation of phosphatidic acid (PA), which facilitates mitochondrial fusion [198]. On the other hand, PA can be converted to diacylglycerol (DAG) on the mitochondrial surface. DAG and Ca^2+^ synergistically activate PKC, leading to phosphorylation of DRP1, terminating the fusion-promoting function of PA. Thus, the interplay between mitoPLD, PKC, and DRP1 may be one of the mechanisms for rapid triggering of division after fusion [6, 198].

#### Mitochondrial fission rapidly follows fusion

Mitochondrial fusion enables the mixing of contents between adjacent mitochondria. Fusion between damaged and healthy mitochondria can help buffer transient stress or defects within the mitochondrial network by diluting toxins [199, 200]. An increase in the GSSG:GSH ratio can promote fusion within minutes [201]. In contrast, during mitophagy, the process from depolarization to entry into lysosomes takes approximately 20 min to several hours [29, 202]. Therefore, mitochondrial fusion is a faster process. Under oxidative stress conditions, fusion can protect mitochondria more rapidly compared to mitophagy.

Given the rapid triggering of asymmetric division after fusion [6, 29, 198], with one daughter mitochondrion often containing a significant amount of damaged material [30], and the short time required for fusion compared to mitophagy, we can hypothesize that: (1) mitochondrial fusion dilutes and buffers damage within the mitochondria through complementation, removing irreversible damage via mitochondrial division followed by mitophagy or other mitochondrial clearance pathways; (2) when transient stresses or defects occur within a portion of mitochondria, the remaining healthy mitochondrial portion may not be sufficient to perform the segregation and division of the damaged portion and, therefore, rely on fusion with other healthy mitochondria; and (3) mitochondrial fusion provides a buffering period for the initiation of mitophagy. These are supported by the findings that ROS generated from mitochondrial damage promotes both mitochondrial fusion and mitophagy through ROS signaling pathways [201, 203]. Mechanistically, Mul1 deficiency increases MFN2 activity, which not only initiates the first phase of mitochondrial hyperfusion, but also antagonizes ER-mito tethering, thereby triggering the second phasic Drp1-dependent mitochondrial fragmentation [191].

#### Opposing roles of Bax/Bak in mitochondrial fission/fusion during homeostasis and apoptosis

During apoptosis, mitochondrial fragmentation that relies on Bax/Bak is likely the result of increased fission and decreased fusion [1, 204]. Mitochondrial fusion prevents apoptosis [205], while apoptosis inhibits mitochondrial fusion. The absence of inner and outer membrane fusion proteins promotes the release of cytochrome C and apoptosis [47], suggesting that inhibition of mitochondrial fusion may exacerbate cell apoptosis. However, the pro-apoptotic proteins Bax/Bak may play opposite roles in regulating mitochondrial morphology in healthy cells compared to their function in cell apoptosis [206]. In line with this, Bax is necessary for maintaining the tetrameric state of mitochondrial fusion proteins during the process of mitochondrial fusion [207]. Overexpression of Mfn2 suppressed the mitochondrial fragmentation observed in Bax/Bak DKO cells, while Drp1 inactivation did not affect mitochondrial fragmentation. This indicates that Bax/Bak can inhibit mitochondrial fission and promote mitochondrial fusion in healthy cells. However, during early stages of apoptosis, Bax/Bak affects ER Ca^2+^ release and subsequently leads to the activation of DRP1-mediated fission [208]. Therefore, both mitochondrial fission and fusion are promoted by Bax/Bak. However, during apoptosis, the two compete with each other, and the increase of mitochondrial fission and decrease of fusion may be attributed to the relocation of Bax/Bak.

### Interactions between mitochondrial dynamics and mitophagy

#### Crosstalk between mitochondrial fission and mitophagy

Mitochondrial fission, the process that separates damaged mitochondria from the mother mitochondria into smaller volumes, may facilitate the clearance of damaged mitochondria through mitophagy. This is supported by findings that inhibition of mitochondrial fission by DRP1 (K38A) or Fis1 RNAi reduces mitophagy [29]. Consistently, mitophagy receptor BNIP3 promotes the division of damaged mitochondria by facilitating the disassembly and release of OPA1 and recruiting DRP1 to the surface of mitochondria [209]. Interestingly, during the ischemic phase of myocardial ischemia/reperfusion injury, sumoylation or Ser616 phosphorylation of DRP1 also contributes to the activation of mitophagy, which in turn promotes mitochondrial membrane potential stabilization and reduces cardiomyocyte apoptosis [210]. These pieces of evidence suggest that mitochondrial fission occurring before mitophagy is beneficial for the clearance of damaged mitochondria. However, during the reperfusion phase, fission is significantly upregulated while the protective mitophagy is suppressed. Reactivating mitophagy can prevent lethal fission [211, 212]. Additionally, the loss of DRP1 enhances Parkin-mediated mitophagy initiation but compromises damage-site selectivity, resulting in indiscriminate mitochondrial removal. This highlights a novel model in which mitochondrial fission does not accelerate mitophagy but instead safeguards the healthy mitochondrial structure against clearance by the Pink1-Parkin mitophagy pathway [8].

Apart from mitochondrial fission, mitochondrial dynamic localization also plays a vital role in mitophagy. For instance, mitochondrial membrane potential stimulates the Pink1-dependent phosphorylation of Miro, facilitating Parkin recruitment, while Parkin ubiquitinates Miro, promoting its degradation and blocking mitochondrial transport [213]. Interestingly, evidence indicates that Miro removal from depolarized mitochondria enhances the clearance of damaged mitochondria by mitophagy [194]. In addition, Miro deficiency triggers mitochondrial clustering around the nucleus, boosting mitophagy in HeLa cells [214]. These findings underscore the intimate interplay between mitochondrial dynamic localization and mitophagy.

#### Crosstalk between mitochondrial fusion and mitophagy

Mitochondrial fusion has been found to be promoted by the overexpression of OPA1, which also decreases the process of mitophagy [29]. In addition, OMA1-mediated cleavage of OPA1 impedes inner membrane fusion, a process before activation of the Pink1/Parkin signaling pathway for mitophagy [215]. These pieces of evidence suggest that mitochondrial fusion and mitophagy are closely interconnected. The mitochondrial fusion protein MFN1/2 is degraded via Pink1-Parkin-mediated ubiquitination during mitophagy when mitochondria are depolarized [90, 216], suggesting that Pink1-Parkin may control the balance between mitochondrial division and fusion [217]. Furthermore, the MYO6 (myosin VI)-Parkin complex prevents damaged mitochondria from fusion again with neighboring mitochondrial populations by forming a physical barrier with F-actin cages [218]. This suggests that the mitochondrial fusion is suppressed by Pink1-Parkin-dependent mitophagy. Consistent with this, in cells with decreased mitochondrial fission and/or increased fusion, mitophagy is diminished [29]. In addition, it has been observed that excessive fusion of mitochondrial networks induced by starvation can protect mitochondria from being eliminated by mitophagy [219]. However, mitophagy can still occur in cells with DRP1 deficiency, despite excessive mitochondrial fusion [107]. This raises doubts on the possibility that mitochondrial fission precedes mitophagy, as fused mitochondria can also be cleared through mitophagy. The prevailing view in this field suggests that mitochondrial fission facilitates mitophagy, while mitochondrial fusion mixes damaged and healthy mitochondria, buffering mitochondrial damage through complementation. However, once the damage to the mitochondria reaches a critical point, fusion alone may not be able to restore them to the normal state. In such cases, mitophagy becomes a crucial mechanism for the clearance of fused mitochondria.

Under conditions of intense stress, such as starvation, calcium overload, oxidative stress, or toxin accumulation, mitochondrial fission may be delayed while fusion increases sharply [219, 220]. This phenomenon is known as stress-induced mitochondrial hyperfusion [35, 221]. A view suggests that fission and fusion serve as two opposing tendencies used to counteract different levels of stress. Increasing fusion/decreasing fission helps overcome low-level stress, while decreasing fusion/increasing fission occurs under high-level stress, such as ischemia/reperfusion, cell death, and mitochondrial depolarization [35]. Although both mitochondrial fusion and mitophagy can respond to mitochondrial damage, fusion is detrimental to mitophagy, as fused mitochondria generally cannot be directly eliminated via mitophagy, because they still contain a healthy part. However, in the presence of excessive damage, fused mitochondria can undergo mitophagy clearance, even though mitophagy signaling degrades mitochondrial fusion-associated proteins, thereby inhibiting fusion.

In summary, mitochondrial fusion is an adaptive repair process for low-level chronic damage. When the damage is too severe to be repaired through mitochondrial fusion or when cells are exposed to acute damage, mitochondrial fission or cell death-related factors may promote mitochondrial division due to decompensation.

### MDV, Pink1/Parkin signaling, and mitochondrial fission

Pink1 regulates the generation of MDVs through a ROS-dependent pathway. Decreased expression of Pink1 results in impaired release of MDVs [126]. Pink1/Parkin mediates the biogenesis of MDVs in the early stage of mitochondrial depolarization, while mitophagy occurs in the later stage [222]. This suggests that the generation of MDVs is the first step in MQC and participates in the selective removal of dispersed mitochondrial parts containing oxidized proteins or lipids to prevent the spreading of damage to the entire organelle. However, the relationship between MDV formation and mitochondrial fission is not clear. In brown adipocytes, heat stimulation increases mitochondrial fission [36] and MDV formation. Interestingly, inhibiting DRP1 activity impaired fission but did not prevent MDV formation or damaged mitochondrial protein sorting [126], whereas promoting DRP1 activity contributes to MDV formation [18]. This suggests the existence of multiple connections between MDV formation and mitochondrial fission. Additionally, in yeasts, Fis1 and Mff, which participate in mitochondrial fission, also exist in peroxisomes. This is consistent with the result showing that DRP1 mediates peroxisome fission in higher eukaryotes [223]. In summary, MDV formation is regulated by Pink1/Parkin signaling and is associated with mitochondrial fission. Considering that MDVs formed by mitochondrial fission can be degraded by peroxisomes, the role of DRP1 in this process needs further investigation.

### The interplay between mitophagy and mitochondrial biogenesis

Mitochondrial biogenesis and mitophagy represent two opposing but coordinated processes that ensure optimal mitochondrial function and cellular health. Mitochondrial biogenesis is triggered in response to various physiological and pathological stimuli, such as nutrient deprivation, physical exercise [224], ischemia preconditioning [225], and other forms of stress known to induce mitophagy [226, 227].

It has been found that PGC-1α/NRF1 promotes mitophagy by regulating the mitophagy receptor FUNDC1 [228]. Consistently, rapid elevation of PGC-1α after ischemic stroke significantly promotes mitophagy via ULK1 [229]. Additionally, overexpression of PGC-1α increases autophagic flux but reduces the co-localization of LC3II and p62 with mitochondria [230]. Increased PGC-1α protein stability as a result of *Park2* gene deletion enhances mitochondrial biogenesis [231]. These findings suggest that PGC-1α promotes both mitochondrial biogenesis and mitophagy and thus plays an important role in mitochondrial turnover.

Mitochondrial biogenesis has complex impacts on mitophagy. A study on aging demonstrated that PGC-1α overexpression reversed the age-related increases of mitophagy markers [232]. PGC-1α overexpression also mitigated the effects of aging on Fis1, MFN2, and DRP1, which may indicate a linkage between mitochondrial biogenesis and the processes of mitochondrial fission, fusion, and mitophagy [232]. A similar effect was observed during cellular reprogramming and mitochondrial remodeling, where inhibition of the mTORC1–PGC1 axis impeded mitochondrial biogenesis [233] while promoting mitophagy [234, 235].

In turn, mitophagy also affects mitochondrial biogenesis, mediated by multiple transcription co-activators and nuclear transcription factors. To maintain mitochondrial homeostasis and sufficient energy supply, damaged mitochondria cleared by mitophagy need to be replaced through mitochondrial biogenesis. Mitophagy activates mitochondrial biogenesis by affecting the NAD^+^:NADH ratio, AMP:ATP ratio, or acetyl-CoA levels [74]. For example, CO activates mitochondrial biogenesis by promoting mitophagy [236]. Mitophagy activates TFEB to promote mitochondrial biogenesis [237]. However, there are exceptions. A previous study found that deletion of the *Park2* gene, which encodes Parkin, a player in mitophagy, elevates mitochondrial biogenesis by increasing PGC-1α protein stability [231]. Therefore, mitophagy and mitochondrial biogenesis generally change synchronously, and mitophagy promotes mitochondrial biogenesis. Nonetheless, the impact of mitochondrial biogenesis on mitophagy is intricate, and mitochondrial biogenesis may suppress mitophagy.

### The ROS, MQC, and ROS loop

ROS can activate mitophagy and promote mitochondrial biogenesis and mitochondrial fusion. ROS can also cause fragmentation of the mitochondrial network. For example, the loss of the ROS regulatory gene *DJ-1* can lead to an increase in stress-induced Parkin recruitment and mitophagy activation [203]. Prdx3, a mitochondrial-specific peroxiredoxin, protects against mitochondrial dysfunction by clearing mitochondrial ROS and may interact with Pink1 to promote mitophagy [238]. Furthermore, ROS can promote mitochondrial biogenesis by activating AMPK, which subsequently activates PGC-1α [82, 239]. Interestingly, mitochondrial biogenesis may reduce the generation of ROS [240]. Thus, in the presence of increased ROS levels, activation of the ROS clearance system may facilitate the clearance of damaged mitochondria via mitophagy and promote mitochondrial biogenesis, thereby reducing ROS levels.

Mitochondrial fusion can be promoted by ROS, and an increase in the GSSG:GSH ratio can facilitate fusion within minutes [201]. However, ROS can also promote mitochondrial fission under certain conditions [241], consistent with the observation that ROS can promote mitophagy. Thus, the net effect of ROS on mitochondrial morphology may depend on the combined action of different signaling pathways activated by ROS, which may be related to the intensity of the ROS signal. However, fragmentation of the mitochondrial network caused by excessive ROS accumulation impedes ROS clearance, and this pathological process defies the homeostatic negative feedback of ROS, in which with the increase of ROS levels, the ROS clearance system is activated, promoting mitochondrial autophagy and mitochondrial fusion, and increasing mitochondrial biogenesis, finally all these reducing the production of ROS.

## Life cycle of a single mitochondrion

**Fig. 5:**
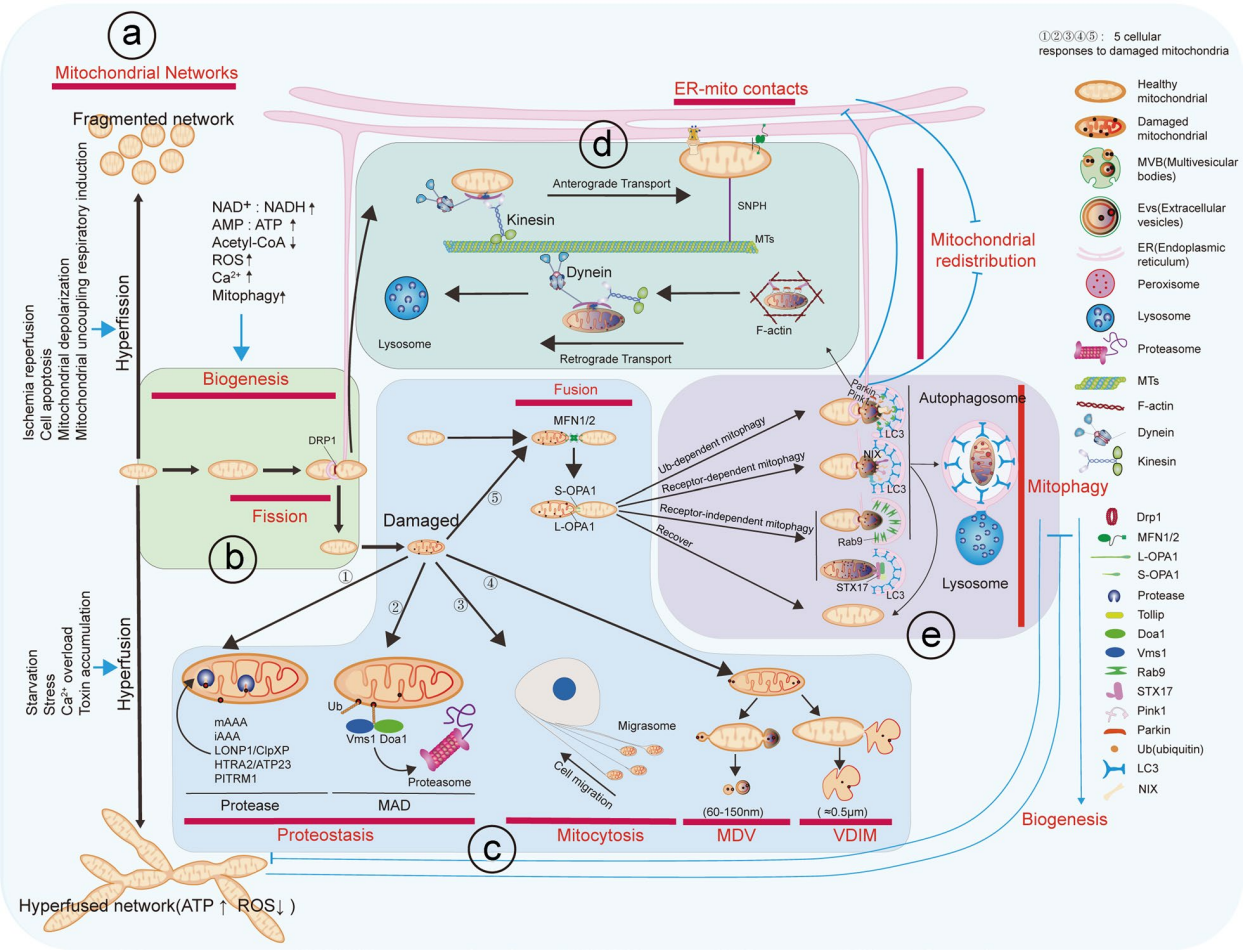
The life cycle of a single mitochondrion. **a** Dynamic changes in mitochondrial networks. Mitochondrial networks change continually, alternating between hyperfusion and fragmentation. This dynamic balance is modulated by various physiological and pathological stimuli, including ischemia–reperfusion, cell apoptosis, mitochondrial depolarization, mitochondrial uncoupling, respiratory induction, starvation, stress, Ca^2+^ overload, and toxin accumulation. Hyperfused networks are associated with increased ATP production and reduced ROS generation. **b** Mitochondrial biogenesis occurs through symmetric division and is influenced by the cellular energy status, indicated by the NAD^+^/NADH ratio, AMP/ATP ratio, and levels of acetyl-CoA, ROS, Ca^2^⁺, and mitophagy activity. **c** Clearance of damaged mitochondria through proteases, proteasomes, transferosomes, MDVs, or mitochondrial fusion, followed by mitophagy as the final defense to maintain mitochondrial homeostasis. **d** Mitochondrial dynamic localization involves anterograde transport primarily mediated by Kinesin, retrograde transport mainly dependent on Dynein, and mitochondrial anchoring through SNPH and ER-mito contacts. **e** Mitophagy, including Ub-dependent mitophagy, receptor-dependent mitophagy, and receptor-independent mitophagy, followed by fusion with lysosomes to form autolysosomes

The lifecycles of individual organelles can be unified and summarized as follows (Fig. 5). First, upon activation of upstream signals such as PGC-1α, a healthy mitochondrion undergoes protein synthesis, assembly, and volume amplification. Occasional abnormalities of protein folding and cleavage will activate the protein quality control system for repair before further steps. Subsequently, the mitochondrion splits in half, and the two small healthy mitochondria are transported to locations of high energy demand. In newly generated mitochondria that execute normal functions, minor protein damage may arise, which activates the protein quality control system to maintain a healthy mitochondrial pool. In the case of more severe damage, including protein damage, mtDNA damage, and lipid peroxidation, damaged mitochondria are removed through MDVs and the mitocytosis pathway.

Second, when these damages accumulate to a level that affects the normal function of a single mitochondrion, mitochondrial fusion is activated to prevent further expansion of damage. For example, if the mitochondrial damage causes an increase in ROS, mitochondrial fusion will be activated first, as it is faster and lasts a shorter time than mitophagy. This process requires the change of L-OPA1 and S-OPA1 ratio that plays an important role in the balance of fission and fusion.

Third, following fusion, the mitochondria undergo sorting via an unknown mechanism that concentrates damaged molecules to one side for uneven mitochondrial fission. The damaged portions are then selectively cleared through mitophagy, with little involvement of migrasomes and MDVs so that healthy mitochondrial portions will not be cleared.

Fourth, in the presence of severe acute damage, mitochondria may directly enter the mitophagy pathway without undergoing mitochondrial fusion and fission or after fusion when the division mechanism is impaired.

Fifth, mitophagy results in an insufficient proportion of mitochondria to support the energy demand. This activates the process of mitochondrial biogenesis, forming a biogenesis-damage-clearance cycle. Additionally, mitochondrial fusion networks are considered to produce more ATP [28] and generate fewer ROS [29]. The diversity of mitochondrial morphology in different tissue cells suggests that mitochondrial fusion and fission are not only a response to damage, but also a response to cellular functional demand [242].

## Conclusion and future directions

Mitochondrial constant self-repair and renewal are carried out by the MQC system. In this review, we provide an overview of various MQC processes, including mitochondrial fusion, fission, dynamic localization, biogenesis, clearance, and ROS clearance, as well as mitochondrial protein quality control mechanisms. Understanding the regulation of the MQC system and the relationships between different regulatory processes can provide a basis for targeted therapies for diseases associated with mitochondrial alterations, including aging, neurodegenerative diseases, cardiovascular diseases, and metabolic diseases.

Healthy and normally functioning mitochondria are essential to meet the energy demand of cell. The MQC system responds to mitochondrial damage and is in charge of the lifecycle of a single mitochondrion. Excessive mitochondrial fission in response to different stresses and damages may result in an excessively fused mitochondrial network or fragmented mitochondrial networks. Mitochondria increase their numbers through mitochondrial biogenesis and newly generated mitochondria are redistributed through microtubule-dependent mitochondrial transport. When damage occurs, mitochondrial homeostasis is maintained through five ways (proteases, proteasomes, transferosomes, MDVs, and mitochondrial fusion), and three mitophagy mechanisms as the final defense.

Although significant progress has been achieved in this field in the recent decades, many questions remain open for further investigation. For instance, the precise way by which mitochondria are transported to their destination remains elusive, requiring further investigations on how the energy need of the cell is converted into signals. The molecules involved in this process could also serve as targets. Moreover, healthy mitochondria can be transferred from one cell to another through TNTs, but it is still not clear whether damaged mitochondria can be transferred to other cells for clearance. Clearance of damaged mitochondria is a prerequisite for normal cellular function and cell survival, especially in highly metabolic cells like neurons. This is carried out by the MQC system through mitophagy, intercellular mitochondrial transfer, MDVs, and mitocytosis. Understanding how these pathways impact mitochondrial function in health and disease is important for further investigation into their mechanisms and therapeutic applications.

It is not yet clear whether MDVs can act as carriers for transporting metabolites between two organelles within a cell or from one cell to another. Moreover, DRP1 mediates peroxisome fission in higher eukaryotes, and MDVs formed by mitochondrial fission can be degraded by peroxisomes. Therefore, it is logical to wonder whether DRP1 plays a role in this process, which needs further study. OMM proteins are important players in mitochondrial-cytoplasmic communication, but how these proteins are targeted and inserted into the OMM is not clear. A recent study revealed that this occurs through topological triaging in the cytosol [243], but this still needs further evaluation. Altogether, future research in this field may open up new avenues for understanding and treating mitochondria-related diseases.

## Data Availability

The data supporting this review are derived from publicly available literature cited in the reference list. No new datasets were generated or analyzed during the current study.

## Abbreviations

MQC: Mitochondrial quality control
ATP: Adenosine triphosphate
ROS: Reactive oxygen species
MDV: Mitochondrial-derived vesicle
Pink1: PTEN-induced kinase 1
TOM: Translocase of the outer mitochondrial membrane
TIM: Translocase of the inner mitochondrial membrane
PARL: Presenilin-associated rhomboid-like protease
c-Pink1: Cleaved Pink1
Miro: Mitochondrial rho GTPase
KIF5: Kinesin family member 5
SNPH: Syntaphilin
NRF1/2: Nuclear respiratory factor ½
PGC-1α: Peroxisome proliferator-activated receptor gamma coactivator 1 alpha
AMPK: AMP-activated protein kinase
GCN5: General control nonrepressed 5
TORC: Transducer of regulated CREB-binding protein
TFEB: Transcription factor EB
CREB: CAMP response element-binding protein
GSK3β: Glycogen synthase kinase 3 beta
PPARs: Peroxisome proliferator-activated receptors
ERRs: Estrogen-related receptors
OPTN: Optineurin
MFN: Mitofusin
OPA1: Optic atrophy 1
L-OPA1: Long isoform of OPA1
S-OPA1: Short isoform of OPA1
CL: Cardiolipin
INF2: Inverted formin-2
Spire1C: Spire-type actin nucleation factor 1C
Miro1: Mitochondrial rho GTPase 1
DRP1: Dynamin-related protein 1
Fis1: Mitochondrial fission 1
Mff: Mitochondrial fission factor
MiD49/51: Mitochondrial dynamics proteins 49/51
Sirt1: Sirtuin 1
PGC1: Peroxisome proliferator-activated receptor gamma coactivator 1
mATG8: Microtubule-associated protein 1 light chain 3
STX17: Syntaxin 17
ULK1: Unc-51 like autophagy activating kinase 1
BECN1: Beclin 1
SNX9: Sorting nexin 9
ATG14: Autophagy-related 14
